# *Blastocystis* infection in Malaysia: Evidence of waterborne and human-to-human transmissions among the Proto-Malay, Negrito and Senoi tribes of Orang Asli

**DOI:** 10.1186/1756-3305-6-40

**Published:** 2013-02-22

**Authors:** Tengku Shahrul Anuar, Mohamed Kamel Abdul Ghani, Siti Nor Azreen, Fatmah Md Salleh, Norhayati Moktar

**Affiliations:** 1 Department of Parasitology and Medical Entomology, Faculty of Medicine, Universiti Kebangsaan Malaysia, Jalan Raja Muda Abdul Aziz, 50300, Kuala Lumpur, Malaysia; 2 Programme of Biomedical Sciences, School of Diagnostic and Applied Health Sciences, Universiti Kebangsaan Malaysia, Jalan Raja Muda Abdul Aziz, 50300, Kuala Lumpur, Malaysia

**Keywords:** *Blastocystis*, Waterborne, Human-to-human, Orang Asli, Malaysia

## Abstract

**Background:**

*Blastocystis* has been described as the most common intestinal parasite in humans and has an increased impact on public health. However, the transmission of this parasite has not been conclusively determined.

**Methods:**

To contribute to a better comprehension of the epidemiology of this infection, a cross-sectional survey aimed at providing the first documented data on the prevalence and risk factors associated with *Blastocystis* infection was carried out among three Orang Asli tribes (Proto-Malay, Negrito and Senoi) in selected villages at Negeri Sembilan, Perak and Pahang, Peninsular Malaysia. Faecal samples were examined by formalin-ether sedimentation and trichrome staining techniques.

**Results:**

Of 500 individuals, 20.4% (102) were detected positive for *Blastocystis*; 13.3% (20/150) of Proto-Malays, 21.6% (30/139) of Negritos and 24.7% (52/211) of Senois were positive for *Blastocystis*, respectively. The positive cases showed a decrease with increasing age and most of the positive cases were observed in individuals less than 15 years old. Multivariate analysis confirmed that drinking untreated water and the presence of other family members infected with *Blastocystis* were significant risk factors of infection among the three tribes and overall population studied.

**Conclusion:**

Essentially, the findings highlighted that *Blastocystis* infection is prevalent among Orang Asli communities in Malaysia. Further studies using molecular approaches to distinguish the subtype of *Blastocystis* is needed. The present study also revealed that this infection may be transmitted through waterborne and human-to-human contact. Therefore, interventions with the provision of clean water supply for the communities and health education especially to the parents are urgently required.

## Background

*Blastocystis* is an anaerobic unicellular eukaryote that can inhabit the intestinal track of humans and many animals, it was first described in 1912 [1]. Numerous cross-sectional surveys have been carried out in different epidemiological settings and hence *Blastocystis* is known to be one of the most frequently found protozoan parasites in human faecal samples, especially in children and adults in developing countries [2,3]. *Blastocystis* prevalence in human often exceeds 5% in industrialized countries and can reach as high as 76% in developing countries [3,4]. However, prevalence data are largely dependent on the methods used for detection. The most common diagnostic technique used worldwide for identification of *Blastocystis* is the permanent stain. The use of xenic cultures in which *Blastocystis* is grown *in vitro* with non-specific microorganisms have been shown to be more sensitive in detecting *Blastocystis* but it is not commonly used in the diagnostic laboratory [5-7]. Several forms of *Blastocystis* are observed in faecal samples include vacuolar, multivacuolar, avacuolar, granular, amoeboid and cyst [2]. However, most laboratories recognize only the vacuolar form as the diagnostic stage since it can be easily distinguished from other protozoa.

Although its role in human disease has been widely debated in the literature during the two last decades, numerous recent *in vivo* and *in vitro* studies strongly suggest that *Blastocystis* is a pathogen [8-10]. Case reports have linked *Blastocystis* infection with various gastrointestinal and extraintestinal symptoms including diarrhea, abdominal pain, depression, fatigue, vomiting, constipation, anorexia, urticaria, skin rash, headaches and flatulence, but this parasite may also play a significant role in several chronic gastrointestinal illnesses such as irritable bowel syndrome and inflammatory bowel disease [11-14]. An epidemiological study on *Blastocystis* showed that the animal handlers have a higher risk of infection and that there is always the possibility of transmission between animals and human [15]. On the other hand, polymerase chain reaction (PCR)-based methodology has demonstrated that there might exist a human-to-human infection among small communities, suggesting that transmission also occurs through the faecal-oral route like other common gastrointestinal parasites [16,17]. Furthermore, transmission can be facilitated by the contamination of the environment, food or water with excreted cysts from the reservoir hosts [18]. Thus, the mode of transmission of the *Blastocystis* has not been conclusively determined [17].

In Malaysia, *Blastocystis* infection has been reported to have a high prevalence among urban and rural communities and also in the water of rivers from recreational areas [19-21]. However, knowledge of the epidemiology and risk factors of *Blastocystis* infection is also rather limited. Therefore, this study was carried out to determine the prevalence of *Blastocystis* and to investigate its association with the socioeconomic characteristics of three Orang Asli (Aborigine) tribes, namely Proto-Malay, Negrito and Senoi in Peninsular Malaysia.

## Methods

### Study area and population surveyed

**Figure 1:**
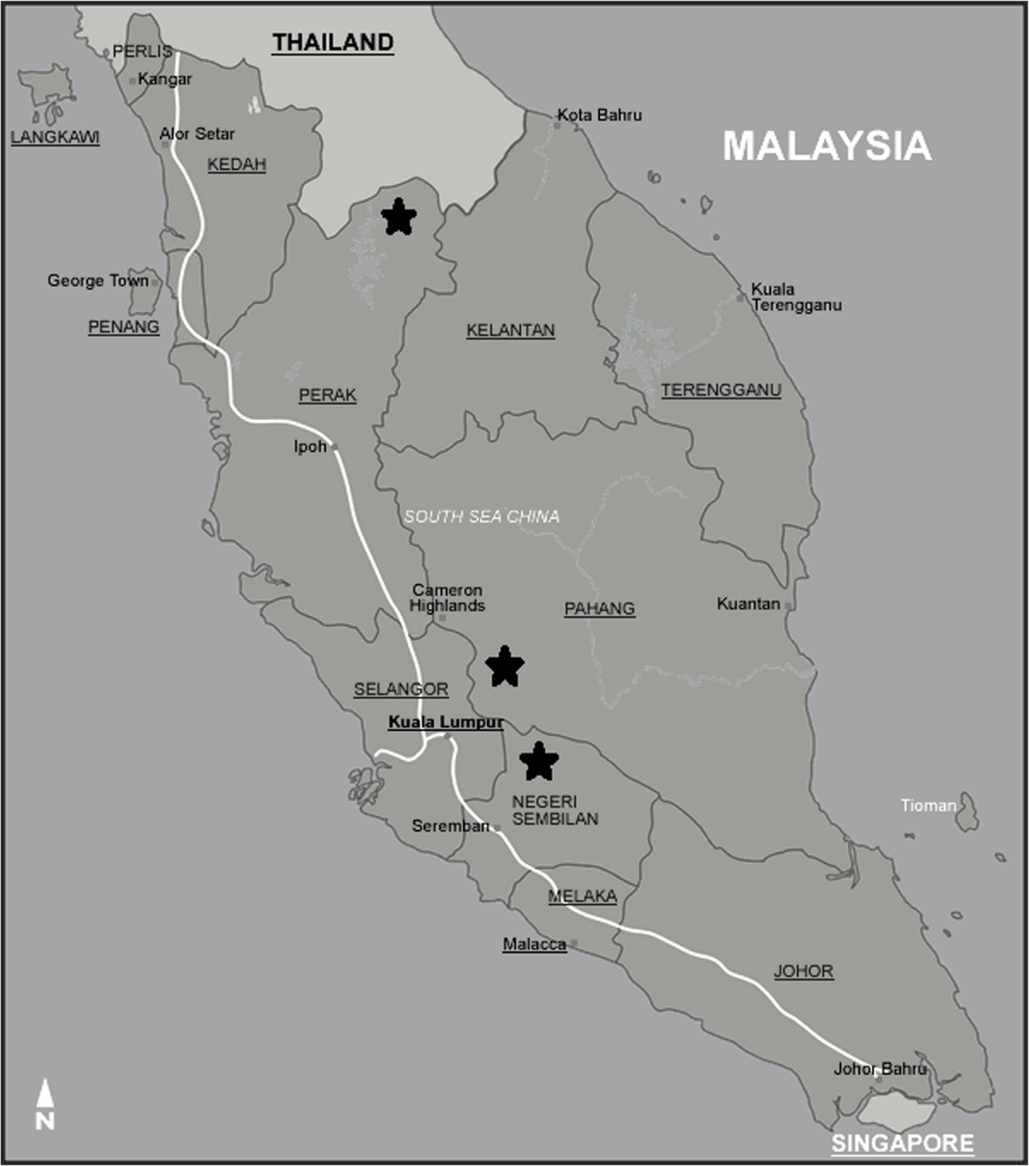
Map showing the location of the villages in Peninsular Malaysia involved in the study (stars).

A series of cross-sectional surveys were conducted in three different states of Peninsular Malaysia in Jelebu (2° 55’ N latitude, 102° 4’ E longitude), Gerik (5° 26’ N latitude, 101° 7’ E longitude) and Temerloh (3° 43’ N latitude, 102° 22’ E longitude) from June to December 2011 (Figure 1). Details of the studied villages and the populations sampled have been described elsewhere [22].

### Sample size

By using the formula provided by Kish [23], the expected sample size was calculated according to the following parameters: expected prevalence of *Blastocystis* at 4.4% and 17% [24,25], confidence interval of 9.5% and absolute precision (d) = 0.05 [26]. The required minimum sample size needed in this study was 65 individuals and the maximum would be 217 individuals in each tribe.

### Questionnaire

A structured questionnaire was developed in English and then translated to Bahasa Melayu (the national language of Malaysia). The questionnaire was pre-tested among Orang Asli individuals who were admitted to Gombak Hospital, Selangor state. Trained research assistants interviewed participants in person, asking questions for demographic data (i.e., age, gender and education level), socioeconomic background (i.e., occupation, household income and educational status), behavioral risks (i.e., personal hygiene such as hand washing and food consumption), environmental sanitation and living condition characteristics (i.e., types of water supply, latrine system, sewage disposal system and presence of domestic animals). Participants were also asked if they had diarrhea and symptoms of gastroenteritis (i.e., vomiting, nausea, abdominal pain, watery stools and blood or mucus stools). For children, the questionnaire was completed by interviewing their parents or the guardian who had given informed consent.

### Field and laboratory procedures: Detection of *Blastocystis*

Following the administration of the questionnaire, a wide mouth screw-capped container pre-labelled with the individual’s name and code was distributed to each participant for the collection of a faecal sample the next day. Their ability to recognize their name was counter-checked. The participant was instructed to scoop a thumb sized faecal sample using a provided scoop into the container. Then, the container was placed in a zip-locked plastic bag. Parents and guardians were instructed to monitor their children during the sample collection in order to ensure that they placed their faecal samples into the correct container. All study participants were asked to provide a sufficiently large faecal sample (at least 10 grams) so that both formalin-ether sedimentation and trichrome staining techniques could be performed. This study had to rely on a single faecal sample collection because of the limitation of resources and the cultural belief of the aborigines against giving of their faecal samples.

**Figure 2:**
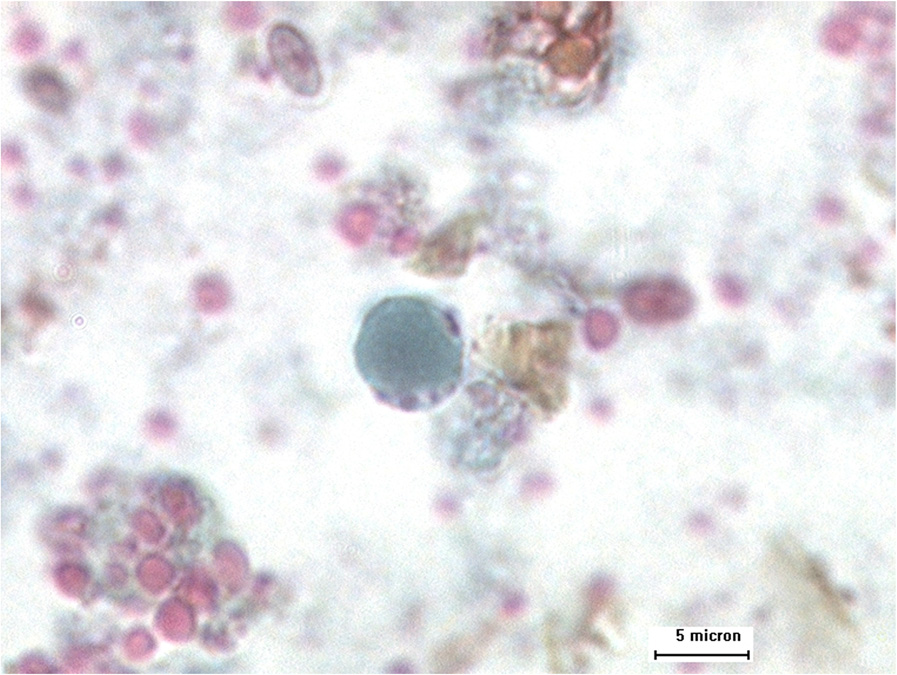
**Vegetative stage of *Blastocystis *sp. (trichrome stain) in faecal sample.**

Faecal samples were processed in the designated area of work in the study village within a maximum of six hours after collection by experienced laboratory technicians. Approximately 5 grams of each faecal sample were kept in a 15 ml centrifuge tube containing 3 ml polyvinyl alcohol (PVA). PVA-fixed samples were forwarded to the parasitological department of the Faculty of Medicine, Universiti Kebangsaan Malaysia. The samples were subjected to trichrome staining. Briefly, the smear cover slip was stained as follows: iodine alcohol (15 minutes), 70% alcohol (10 minutes), trichrome stain (10 minutes), acid alcohol (3 seconds), 95% alcohol (5 minutes), absolute alcohol (5 minutes) and wintergreen oil (5 minutes) [27]. The cover slip was mounted using DPX and examined under a light microscope at magnifications of 1000x. Additionally, another half of the samples were kept unfixed and stored at 4°C upon arrival at the laboratory for further analysis using formalin-ether sedimentation. Briefly, 2 grams of faecal sample were mixed with 7 ml of formalin and 3 ml of ether, centrifuged, stained with Lugol’s iodine and finally examined under a light microscope at magnifications of 400x [28]. The sample was reported as positive if cysts and/or vacuolar forms of *Blastocystis* were detected by any of these two techniques (Figure 2).

### Data management and analysis

Data was entered into a Microsoft Access and was cross-checked by the technical staff in order to ensure that data were entered correctly. Statistical analysis was performed using the SPSS version 20 (SPSS, Chicago, IL, USA). Only those individuals who had formalin-ether sedimentation and trichrome staining results together with complete questionnaire data were included in the final analyses.

For descriptive analysis, rate (percentage) was used to describe the characteristics of the studied population, including the prevalence of *Blastocystis*. A Chi-squares test (*χ*^2^) was used to test the associations between the variables. In the univariate analysis, the dependent variable was prevalence of *Blastocystis*, while the independent variables were demographic (gender and age group) and socioeconomic factors, behavioral risks, environmental sanitation, living condition characteristics and gastrointestinal symptoms. All variables that were significantly associated with the prevalence of *Blastocystis* in the univariate model were included in a logistic regression analysis to identify the risk factors for *Blastocystis* infection with control for the effects of possible confounders. For each statistically significant factor, an odds ratio (OR) and 95% confidence interval (CI) were computed by the univariate and multivariate logistic regression analyses. The level of statistical significance was set as *p* < 0.05. Furthermore, Mantel-Haenszel Chi-square test was used to test whether there is any confounding effect of the contingency tables.

### Ethical issues

Prior to data collection, the study protocol (Reference Number: UKM 1.5.3.5/244/FF-165-2011) was reviewed and approved by the Ethics Committee of Universiti Kebangsaan Malaysia Medical Centre (UKMMC) and permission for field work was obtained from the Department of Orang Asli Development (JAKOA). Village meetings were held and village authorities and villagers were handed detailed explanations of the aims, procedures, potential risks and benefits of the study. During the meeting, they were also informed that their identity and personal information would be kept strictly confidential, and they could withdraw from the study at any point of time without citing reasons for doing so. If they agreed to participate, their consent was obtained in written form signature (or thumbprint for those who were illiterate) or parents were approached for consent on behalf of their children.

## Results

### Demographic and socioeconomic profiles

A total of 500 individuals aged from 2 to 74 years participated in this study with a median age of 18 years [interquartile range (IQR) 9–35]; and consisted of 150 (30%) Proto-Malays, 139 (28%) Negritos and 211 (42%) Senois.

Overall, the studied population came from a low socioeconomic background with more than half having less than 6 years of formal education. A high percentage of household income of less than RM500.00 (US$ 162.42) was predominantly seen in the Negrito (82.7%) and Senoi (60.7%) tribes. Most (95.3%) houses in Proto-Malay villages had piped water supplies and toilet facilities; conversely piped water supplies and safe sewage disposal facilities were lacking in the villages of the Negrito and Senoi tribes. General characteristics of each tribe, including their demographic and socioeconomic profiles are presented in Table 1.

**Table 1:** General characteristics of the Orang Asli communities that participated in this study

**Characteristics/Tribes**	**Overall**	**Proto-Malay**	**Negrito**	**Senoi**
	n (%)	n (%)	n (%)	n (%)
*Age groups (years)*				
<15	221 (44.2)	59 (39.3)	72 (51.8)	90 (42.7)
≥15	279 (55.8)	91 (60.7)	67 (48.2)	121 (57.3)
*Gender*				
Male	219 (43.8)	66 (44.0)	66 (47.5)	87 (41.2)
Female	281 (56.2)	84 (56.0)	73 (52.5)	124 (58.8)
*Socioeconomic status*				
Father’s education (<6 years)	222 (74.0)	54 (66.7)	68 (77.3)	100 (76.3)
Mother’s education (<6 years)	232 (56.2)	53 (65.4)	77 (87.5)	102 (77.9)
Low monthly household income (<RM500)	260 (52.0)	17 (11.3)	115 (82.7)	128 (60.7)
Working mothers	153 (51.0)	45 (55.6)	33 (37.5)	75 (57.3)
Large family (≥8 members)	171 (34.2)	32 (21.3)	63 (45.3)	76 (36.0)
Supplied with piped water	357 (71.4)	143 (95.3)	76 (54.7)	138 (65.4)
Presence of toilet at household)	308 (61.6)	143 (95.3)	48 (34.5)	117 (55.5)

RM = Malaysian Ringgit; (US$100 = RM307.85) [1^st^ February 2013].

n = Number examined.

### Prevalence and distribution of *Blastocystis* infection

The prevalence and distribution of *Blastocystis* infection are shown in Table 2. The overall prevalence of this infection was 20.4%. It is evident that, of 139 individuals studied from the Negrito tribe, 21.6% were infected with *Blastocystis*. However, individuals from the Senoi tribe had a relatively high prevalence of this infection which was 24.7%, whereby only 13.3% of the Proto-Malays reported positive for *Blastocystis*. Overall, the positive cases decreased with increasing age and most of the positive cases were observed in individuals of less than 15 years old. Similar findings were observed in the Proto-Malay, Negrito and Senoi tribes. The overall prevalence of *Blastocystis* was higher in females, with similar observations seen in all tribes. However, there was no significant difference of this infection between genders. With regards to tribe, it was observed that the Senoi presented a greater risk of *Blastocystis* infection than the Negrito and Proto-Malay tribes. On the other hand, there was no significant difference in the prevalence of *Blastocystis* infection among these three tribes.

**Table 2:** **Prevalence of *Blastocystis *infection among different Orang Asli tribes according to age groups and gender**

	**Overall**			**Proto-Malay**			**Negrito**			**Senoi**		
	n	n	%	n	n	%	n	n	%	n	n	%
	positive	tested	positive	positive	tested	positive	positive	tested	positive	positive	tested	positive
*Age groups (years)*												
<15	50	221	22.6	10	59	17.0	16	72	22.2	24	89	27.0
≥15	52	279	18.6	10	91	11.0	14	67	20.9	28	122	23.0
*Gender*												
Male	41	219	18.7	8	66	12.1	14	66	21.2	19	87	21.8
Female	61	281	21.7	12	84	14.3	16	73	21.9	33	124	26.6
Total	102	500	20.4	20	150	13.3	30	139	21.6	52	211	24.7

n = Number examined.

### Co-infection with other intestinal parasites

For all positive faecal samples with *Blastocystis* (n = 102), 82.4% showed co-infection with one or more other parasites. *Trichuris trichiura* (53.9%) was the most common intestinal parasite found in conjunction with *Blastocystis*, followed by *Ascaris lumbricoides* (29.4%), *Entamoeba histolytica/dispar/moshkovskii* (26.5%), *Entamoeba hartmanni* (22.6%), *Entamoeba coli* (20.6%), *Giardia intestinalis* (17.7%), *Iodamoeba butschlii* (13.7%), *Endolimax nana* (6.9%), hookworm (5.9%) and *Chilomastix mesnilii* (2%). Furthermore, twelve samples were isolated from symptomatic subjects.

### Risk factors for *Blastocystis* infection

Univariate analysis identified drinking untreated water (OR = 2.92; 95% CI = 1.84, 4.63; *p* < 0.001), not washing hands after playing with soil or gardening (OR = 1.58; 95% CI = 1.02, 2.45; *p* = 0.040) and the presence of other family members infected with *Blastocystis* (OR = 8.21; 95% CI = 5.07, 13.30; *p* < 0.001) as the main risk factors for the overall population studied (Table 3). In Proto-Malays, drinking untreated water (OR = 6.25; 95% CI = 1.52, 25.70; *p* = 0.019) and the presence of other family members infected with *Blastocystis* (OR = 21.48; 95% CI = 6.70, 68.82; *p* < 0.001) were identified as risk factors for *Blastocystis* (Table 4). In Negritos, drinking untreated water (OR = 2.79; 95% CI = 1.10, 7.03; *p* = 0.026) and the presence of other family members infected with *Blastocystis* (OR = 6.19; 95% CI = 2.55, 14.98; *p* < 0.001) were the predictors for *Blastocystis* infection (Table 4). Three risk factors were found to be positively significant in Senois; drinking untreated water (OR = 2.35; 95% CI = 1.10, 5.03; *p* = 0.025), not washing hands after playing with soil or gardening (OR = 2.00; 95% CI = 1.06, 3.78; *p* = 0.031) and the presence of other family members infected with *Blastocystis* (OR = 6.11; 95% CI = 3.10, 12.03; *p* < 0.001) (Table 4).

**Table 3:** **Potential risk factors associated with *Blastocystis *infection among the overall population studied (univariate analysis, n = 500)**

**Variables**	**No. examined**	**Infected (%)**	**OR (95% CI)**	***p*-value**
Age (years)				
<15	221	22.6	1.28 (0.83,1.97)	0.272
≥15	279	18.6	1	
Gender				
Female	281	21.7	1.20 (0.77,1.87)	0.411
Male	219	18.7	1	
Drinking untreated water				
Yes	232	29.4	2.92 (1.84,4.63)	<0.001^a,b^
No	265	12.5	1	
Bathing and washing in the river				
Yes	143	23.8	1.33 (0.83,2.12)	0.236
No	357	19.0	1	
Not washing hands after playing with soil or gardening				
Yes	197	25.0	1.58 (1.02,2.45)	0.040^a^
No	303	17.4	1	
Presence of domestic animals				
Yes	309	20.1	0.96 (0.62,1.49)	0.862
No	191	20.8	1	
Indiscriminate defecation				
Yes	192	22.9	1.28 (0.82,1.99)	0.270
No	308	18.8	1	
Sewage disposal				
Outdoor	240	22.1	1.22 (0.79,1.87)	0.369
Common drainage	260	18.8	1	
Eating with hands				
Yes	328	20.2	0.96 (0.60,1.52)	0.859
No	172	20.9	1	
Consuming raw vegetables				
Yes	238	21.1	1.08 (0.70,1.68)	0.717
No	262	19.8	1	
Eating fresh fruits				
Yes	395	18.4	0.63 (0.39,1.01)	0.055
No	105	26.4	1	
Father’s education				
Non-educated (<6 yrs)	222	24.3	1.47 (0.76,2.83)	0.247
Educated (>6 yrs)	78	17.9	1	
Mother’s education				
Non-educated (<6 yrs)	232	25.0	1.93 (0.93,4.03)	0.075
Educated (>6 yrs)	68	14.7	1	
Working mothers				
Yes	153	23.8	1.15 (0.67,1.97)	0.625
No	147	21.5	1	
Household members				
≥8	171	21.6	1.12 (0.71,1.77)	0.621
<8	329	19.8	1	
Household monthly income				
<RM500	260	23.5	1.49 (0.96,2.32)	0.077
>RM500	240	17.1	1	
Other family members infected with *Blastocystis*				
Yes	116	50.9	8.21 (5.07,13.30)	<0.001^a,b^
No	384	11.2	1	

RM = Malaysian Ringgit; (US$100 = RM307.85) [1^st^ February 2013].

Reference group marked as OR = 1 [OR = Odds Ratio].

CI = Confidence interval.

^a^ Significant association (*p* < 0.05).

^b^ Variables were confirmed by multivariate analysis as significant risk factors.

**Table 4:** **Associations between *Blastocystis *infection and risk factors among different Orang Asli tribes**

	**Proto-Malay (n = 150)**				**Negrito (n = 139)**				**Senoi (n = 211)**			
**Variables**	**No. examined**	**Infected (%)**	**OR**	***p*-value**	**No. examined**	**Infected (%)**	**OR**	***p*-value**	**No. examined**	**Infected (%)**	**OR**	***p*-value**
			**(95% CI)**				**(95% CI)**				**(95% CI)**	
*Age (years)*												
<15	59	16.9	1.65 (0.64,4.26)	0.294	72	22.2	1.08 (0.48,2.43)	0.849	90	26.7	1.21 (0.64,2.67)	0.557
≥15	91	11.0	1		67	20.9	1		121	23.1	1	
*Gender*												
Female	84	14.3	1.21 (0.46,3.15)	0.699	73	21.9	1.04 (0.46,2.34)	0.920	124	26.6	1.30 (0.68,2.48)	0.428
Male	66	12.1	1		66	21.2	1		87	21.8	1	
*Drinking untreated water*												
Yes	9	44.4	6.25 (1.52,25.70)	0.019^a,b^	79	28.0	2.79 (1.10,7.03)	0.026^a,b^	144	29.2	2.35 (1.10,5.03)	0.025^a,b^
No	141	11.3	1		60	12.3	1		67	14.9	1	
*Bathing and washing in the river*												
Yes	7	14.3	1.09 (0.12,9.54)	0.939	63	28.6	2.13 (0.94,4.86)	0.068	73	20.5	0.71 (0.36,1.40)	0.315
No	143	13.3	1		76	15.8	1		138	26.8	1	
*Not washing hands after playing with soil or gardening*												
Yes	27	11.5	0.82 (0.22,3.04)	0.767	91	22.0	1.07 (0.46,2.52)	0.876	79	32.9	2.00 (1.06,3.78)	0.031^a^
No	123	13.7	1		48	20.8	1		132	19.7	1	
*Presence of domestic animals*												
Yes	120	14.2	1.49 (0.41,5.44)	0.548	70	16.7	0.61 (0.26,1.45)	0.261	119	27.7	1.45 (0.77,2.81)	0.237
No	30	10.0	1		69	24.7	1		92	20.7	1	
*Indiscriminate defecation*												
Yes	7	14.3	1.09 (0.12,9.54)	0.939	91	23.1	1.30 (0.54,3.11)	0.555	94	23.4	0.89 (0.47,1.67)	0.708
No	143	13.3	1		48	18.8	1		117	25.6	1	
*Sewage disposal*												
Outdoor	21	9.5	0.65 (0.14,3.03)	0.580	106	21.7	1.03 (0.40,2.67)	0.953	113	24.8	1.02 (0.54,1.90)	0.961
Common drainage	129	14.0	1		33	21.2	1		98	24.5	1	
*Eating with hands*												
Yes	70	12.0	0.79 (0.31,2.04)	0.631	105	22.4	1.25 (0.46,3.40)	0.657	153	22.6	0.67 (0.34,1.32)	0.247
No	80	14.7	1		34	18.8	1		58	30.4	1	
*Consuming raw vegetables*												
Yes	63	12.7	0.91 (0.35,2.37)	0.846	113	24.6	3.74 (0.83,16.89)	0.068	62	23.1	0.88 (0.46,1.76)	0.724
No	87	13.8	1		26	8.0	1		149	25.3	1	
*Eating fresh fruits*												
Yes	129	14.7	3.46 (0.44,27.28)	0.213	122	19.7	0.45 (0.15,1.34)	0.142	144	21.0	0.62 (0.33,1.17)	0.139
No	21	4.8	1		17	35.3	1		67	29.9	1	
*Father’s education*												
Non-educated (<6 yrs)	54	14.8	1.00 (0.27,3.67)	1.000	68	26.5	2.04 (0.53,7.79)	0.290	100	28.0	1.33 (0.52,3.44)	0.551
Educated (>6 yrs)	27	14.8	1		20	15.0	1		31	22.6	1	
*Mother’s education*												
Non-educated (<6 yrs)	53	18.9	3.02 (0.61,14.89)	0.158	77	22.1	0.50 (0.13,1.90)	0.298	102	30.4	2.73 (0.88,8.50)	0.075
Educated (>6 yrs)	28	7.1	1		11	36.4	1		29	13.8	1	
*Working mothers*												
Yes	45	15.6	1.14 (0.33,3.95)	0.834	33	25.8	1.18 (0.43,3.25)	0.753	75	28.0	1.17 (0.53,2.56)	0.701
No	36	13.9	1		55	22.8	1		56	25.0	1	
*Household members*												
≥8	32	12.5	0.91 (0.28,2.94)	0.876	63	25.4	1.51 (0.67,3.39)	0.320	76	22.4	0.82 (0.42,1.60)	0.565
<8	118	13.6	1		76	18.4	1		135	25.9	1	
*Household monthly income*												
<RM500	17	17.6	1.46 (0.38,5.62)	0.578	115	24.3	3.54 (0.78,16.01)	0.083	128	23.4	0.85 (0.45,1.60)	0.613
>RM500	133	12.8	1		24	8.3	1		83	26.5	1	
*Other family members infected with Blastocystis*												
Yes	18	61.1	21.48 (6.70,68.82)	<0.001^a,b^	33	48.5	6.19 (2.55,14.98)	<0.001^a,b^	65	49.2	6.11 (3.10,12.03)	<0.001^a,b^
No	132	6.8	1		106	13.2	1		146	13.7	1	

RM = Malaysian Ringgit; (US$100 = RM307.85) [1^st^ February 2013].

Reference group marked as OR = 1 [OR = Odds Ratio].

CI = Confidence interval.

^a^ Significant association (*p* < 0.05).

^b^ Variables were confirmed by multivariate analysis as significant risk factors.

Logistic regression analysis of the overall population studied again confirmed that drinking untreated water (OR = 2.75; 95% CI = 1.66, 4.55; *p* < 0.001) and the presence of other family members infected with *Blastocystis* (OR = 7.96; 95% CI = 4.84, 13.08; *p* < 0.001) as the real risk factors for *Blastocystis* infection. Similar observations were also identified in the Proto-Malay, Negrito and Senoi tribes.

## Discussion

The epidemiological studies on *Blastocystis* infection have been inconclusive in several aspects, particularly regarding the source of infection and mode of transmission [29,30]. Several studies have indicated human-to-human and animal-to-animal transmission models and it has been revealed that the cyst form was the only transmissible stage of this parasite [31,32]. A recent study by Lee *et al.*[33] provided molecular evidence supporting zoonotic transmission of *Blastocystis* in a rural community in Nepal. On the other hand, the prevalence of *Blastocystis* infection might be varied due to the laboratory method used for detection, age group and hygienic condition of the population studied [34]. However, high prevalence reported from developing countries confirmed that poor hygiene was involved in the transmission of the disease.

In the present study, the overall prevalence of *Blastocystis* among Orang Asli in Malaysia was 20.4%, which differs from previous local reports [19,24,35,36] but in agreement with the latest report by Abdulsalam *et al.*[21]. Comparing our findings with studies from other countries showed that the prevalence reported by the present study was similar to those reported in Jordan (25%), Brazil (26.5%), Colombia (22.4%) and Nepal (26.1%) [37-40]. However, the prevalence rate of the infections observed in this study is higher than reported in Thailand and India [34,41]. In contrast, considerably higher prevalence rates of 31%, 48.7%, 32% and 40.7% were reported among Egyptians, Filipinos, Pakistanian and Argentinean individuals, respectively [12,42-44]. Our findings showed no significant difference in the prevalence of *Blastocystis* infection according to age and gender of the participants, and this is consistent with the results of previous reports [18,19,21,40]. However, a study carried out by Li *et al.*[45] demonstrated that individuals aged 60 years and above had the highest prevalence of *Blastocystis* in Shanghai municipality and Yangjia country, which is similar to a finding reported from Glasgow [7]. Furthermore, Al-Fellani *et al.*[46] reported males were more frequently infected (28.98%) with *Blastocystis* than females (23.89%) and the difference was statistically significant. Likewise, several studies also reported significantly higher prevalence in male than female subjects [37,47].

This present study demonstrated that the prevalence rates of this infection were higher in Negritos and Senois than the Proto-Malay tribe. This could be attributed to the lack of basic amenities in the Negrito and Senoi tribes where almost 50% of the communities are not accessed to piped water supply and safe toilet facilities. It indicates that the poor provision of basic amenities plays an important role in the transmission of *Blastocystis* in Senois and Negritos. Similar findings were seen in *Entamoeba histolytica/dispar/moshkovskii* infection carried out in the same communities which reported a high prevalence rate of this infection among members of the Negritos who have poor housing conditions and basic amenities [48]. However, this finding contradicted previously reported prevalence of *Giardia intestinalis* infection carried out in the same communities which demonstrated a high prevalence rate of giardiasis among members of the Proto-Malay tribe who have a better housing condition and basic amenities [22]. Other family members infected with giardiasis were identified as a risk factor of the infection and human-to-human transmission was postulated as a main mode of transmission of giardiasis in the communities [22].

Waterborne transmission has been speculated as the mode of transmission of *Blastocystis* in several studies, especially those conducted in tropical countries and in travelers who just returned from these countries [37,49]. These travelers also gave a history of drinking untreated water while they were abroad [49]. On further analysis of risk factors using multivariate analysis, we found that those who drink untreated water or unboiled water were statistically significant for the Proto-Malay (*p* = 0.019), Negrito (*p* = 0.026) and Senoi tribes (*p* = 0.025). Moreover, drinking untreated water was also found to be a significant predictor of acquiring *Blastocystis* infection in the overall population studied (*p* < 0.001). It is interesting to note that although the prevalence rate of *Blastocystis* was low in Proto-Malays as compared to Senois and Negritos, and almost 95% of the population in Proto-Malay villages have access to piped water supply, individuals from the Proto-Malay tribe who drink untreated water or unboiled water have a higher risk of acquiring this infection as compared to individuals in Senoi and Negrito tribes. During the visits to the villages, we observed that most of the Proto-Malays preferred to drink unboiled water because it was tastier than boiled water. The cysts of *Blastocystis* are rather small compared to the cysts of other common protozoa such as *Giardia intestinalis* and *Entamoeba histolytica*[29]. Thus, it is likely that the cysts of *Blastocystis* could escape the conventional water filtration techniques [29]. Furthermore, cysts of *Blastocystis* were resistant to chlorine at the standard concentration used in tap water [50] and able to survive in water for up to 19 days at a normal temperature, but are fragile at extremes of heat and cold, and in common disinfectants [51,52]. All these characteristics made untreated water a suitable source of infection of this protozoa. Besides that, post-treatment contamination of drinking water that is usually stored in a bucket for household use and the common practice of drinking unboiled water might be the possible reason for the high risk. The unboiled or unfiltered drinking water might have been contaminated with *Blastocystis* cysts from faecal shedding, which may lead to *Blastocystis* infection as suggested by a group in Thailand and Nepal [18,40]. During the visit to the villages, we observed that 28.6% of the households from the three tribes used water from the wells, streams and rain with or without further treatment by filtration or boiling. Therefore, the present study provides baseline data for the role of waterborne transmission in *Blastocystis* infection among the Proto-Malay, Negrito and Senoi tribes. However, there is a need for further investigations to assess whether the reported association of *Blastocystis* infection and drinking untreated water is a causal association.

It is interesting to note that in the present study, the presence of other family members infected with *Blastocystis* has been identified as the significant risk factors (*p* < 0.001) among Proto-Malays, Negritos, Senois and overall population, respectively. This evidence suggests infection can occur within families and human-to-human transmission of *Blastocystis* is another possible mode of transmission identified in the present study. Sharing of poorly maintained sanitary facilities with a household may facilitate human-to-human transmission [40]. Therefore, maintaining clean sanitary facilities and practices are important to avoid transmission of *Blastocystis* to others. Furthermore, screening of other family members should also be recommended as one of the strategies in controlling *Blastocystis* infection in all tribes of Orang Asli communities as an infected family member appears to be an important risk factor for this infection. A family outbreak of *Blastocystis* infection with gastroenteritis has been reported [53]. Thus, the possibility of a *Blastocystis* outbreak is of concern. Likewise, a study done by Pipatsatitpong *et al.*[54] demonstrated that orphans who lived in the same room as childcare workers who were infected with *Blastocystis* had a higher risk of acquiring blastocystosis than those who lived in the rooms without infected childcare workers. Transmission of *Blastocystis* has also been reported among family members [55,56] and among mentally deficient persons in institutions [57,58].

In the present study, we have been using both concentration technique and trichrome staining. Some authors suggested that concentration techniques were unsuitable to detect *Blastocystis* from faecal samples because the parasite could be easily disrupted [59]. In contrast, several other studies have reported that concentration techniques are useful [60,61]. Our results showed that trichrome stain was suitable for the diagnosis of *Blastocystis* infection. It is noted that all other reports on the prevalence of *Blastocystis* in Malaysia used different method which was *in vitro* cultivation. However, cultivation of the organism plays a minor role especially as results are available only after 48 to 72 hours [62]. This may not make them appropriate for comparison with this study. Nonetheless, these results could serve as baseline information on the prevalence and risk factors of *Blastocystis* among Orang Asli in Malaysia.

The present study has several limitations. Firstly, the findings were based on formalin-ether sedimentation and trichrome staining techniques. We were unable to culture in Jones’ medium due to limited faecal samples. By virtue of this fact, if *in vitro* cultivation was employed as a diagnostic tool, the prevalence of *Blastocystis* may be higher than the current reported prevalence due to its sensitivity. Secondly, the inconsistency between observation and response given by the subjects. These inconsistencies could be caused by differences in educational background among the Orang Asli.

## Conclusion

In conclusion, this is the first study to comprehensively provide the prevalence and risk factors of *Blastocystis* infection among the Proto-Malay, Negrito and Senoi tribes. Our postulation concurs with the study carried out previously which reported that the transmission to humans was through drinking untreated water. Furthermore, the present study also observes human-to-human transmission appears to be one of the modes of transmission of *Blastocystis* in these communities. Therefore, it is suggested that an intervention strategy should be taken to protect the community and to advise them to drink boiled water. Screening and giving treatment to the infected family members on the basis of one affected member would appear to be justified in order to avoid or reduce the *Blastocystis* infection among Orang Asli.

## Competing interests

The authors’ declare that they have no competing interests.

## Authors’ contributions

TSA was involved in all phases of the study, including study design, data collection, data analysis and write up of the manuscript; NM and MKAG supervised the study, and revised the manuscript; NM and MKAG were involved in the statistical analysis of data; SNA and FMS were involved in the collection and laboratory examination of samples. All authors read and approved the final manuscript. TSA and NM are the guarantors of the paper.

## Financial support

This work was supported in part by the UKMMC Fundamental Research Grant (FF-165-2011) and Special Research University Grant (UKM-GUP-2011-316) from Universiti Kebangsaan Malaysia.
